# Trial characteristics, geographic distribution, and selected methodological issues of 1425 infertility trials published from 2012 to 2023: a systematic review

**DOI:** 10.1093/hropen/hoaf004

**Published:** 2025-01-24

**Authors:** Qian Feng, Wanlin Li, James Crispin, Salvatore Longobardi, Thomas D’Hooghe, Ben W Mol, Wentao Li

**Affiliations:** Department of Obstetrics and Gynaecology, Monash University, Clayton, VIC, Australia; Department of Obstetrics and Gynaecology, Monash University, Clayton, VIC, Australia; Department of Obstetrics and Gynaecology, Monash University, Clayton, VIC, Australia; Global Clinical Development Fertility, Research and Development, Merck, Darmstadt, Germany; Research Group Reproductive Medicine, Department of Development and Regeneration, Organ Systems, Group Biomedical Sciences, KU Leuven (University of Leuven), Leuven, Belgium; Department of Obstetrics, Gynecology and Reproductive Sciences, Yale School of Medicine, New Haven, CT, USA; Global Medical Affairs Fertility, Research and Development, Merck Healthcare KGaA, Darmstadt, Germany; Department of Obstetrics and Gynaecology, Monash University, Clayton, VIC, Australia; Aberdeen Centre for Women’s Health Research, School of Medicine, Medical Sciences and Nutrition, University of Aberdeen, Aberdeen, UK; National Perinatal Epidemiology and Statistics Unit (NPESU), Centre for Big Data Research in Health, School of Clinical Medicine, Faculty of Medicine, University of New South Wales, Sydney, NSW, Australia

**Keywords:** randomized controlled trials, infertility, research quality, trial registration, outcome reporting, *in vitro* fertilization, assisted reproduction, live birth, primary outcomes

## Abstract

**STUDY QUESTION:**

What are the trial characteristics, geographic distribution, and selected methodological issues of randomized controlled trials (RCTs) in infertility published from 2012 to 2023?

**SUMMARY ANSWER:**

Of the 1425 infertility RCTs, over two-thirds focused on IVF, nearly two-fifths did not use pregnancy or live birth as the primary outcome, a third lacked a primary outcome, a half were unregistered, and just over half were conducted in China (22%), Iran (20%), or Egypt (10%).

**WHAT IS KNOWN ALREADY:**

RCTs are the main source of evidence on the effectiveness of interventions. Knowledge about RCTs in infertility from the recent past will help to pinpoint research gaps and prioritize the future research agenda. Here, we aim to present a descriptive analysis of trial characteristics, geographic distribution, and selected methodological issues in infertility trials published in the last decade.

**STUDY DESIGN, SIZE, DURATION:**

This is a systematic review. We systematically searched Embase, Medline, and Cochrane Central for RCTs in infertility from January 2012 to August 2023. RCTs involving subfertile women and women who reported pregnancy endpoints were eligible, while conference abstracts or secondary analyses were not. We did not limit our search based on the language of the articles.

**PARTICIPANTS/MATERIALS, SETTING, METHODS:**

The full articles were text-mined and manually extracted for the description of trials’ characteristics (e.g. sample size, blinding method, types of intervention), the country where the patients were recruited, and methodological issues (trial registrations and specification of primary outcomes). We extracted funding statements from Dimensions, a literature database chosen for its comprehensive and robust metadata. Gross domestic product (GDP) data were obtained from the United Nations’ official website. The accuracy of extracted data was validated in a random sample of 50 articles, and false positivity and false negativity were all at or below 8%. We used descriptive statistics, including frequencies and percentages to illustrate the overall and temporal trends.

**MAIN RESULTS AND THE ROLE OF CHANCE:**

Among 8757 records, we found 1425 eligible RCTs, with a median sample size of 140, and 33.3% had a sample size <100. Most (69.6%) of the trials focused on IVF, with the rest focusing on ovulation induction (12.4%), intrauterine insemination (10.6%), surgeries (4.8%), or other interventions (2.6%). Regarding the geographic distribution, China (n = 310), Iran (n = 284), and Egypt (n = 138) contributed to 51% of the RCTs, followed by Turkey (n = 82), India (n = 71), and the USA (n = 69); mainland Europe produced 343 trials. Ranked by publications of trials per trillion GDP, Greece had the most papers with 4.6, followed by Iraq at 3.9, and Iran at 2.5. Regarding trial registration, 47.8% of trials were unregistered, the proportion of studies that were unregistered halved from 70.0% in 2012 to 34.6% in 2022. Of all RCTs, 37.6% had primary outcomes unspecified; the proportion of trials specifying primary outcomes increased from 49.5% in 2012 to 61.4% in 2022. The proportion of trials which declared receiving no funding was 76.9%.

**LIMITATIONS, REASONS FOR CAUTION:**

We primarily used text mining for data extraction. Despite optimizing the algorithm to identify all outcome definitions and manually curating the extracted data, there were inaccuracies in data extraction; however, the false positivity and false negativity of data extraction were all at or below 8%. Also, we focused on trials reporting pregnancy outcomes, as these are of primary interest to patients and carry significant implications on clinical practice. However, we acknowledge that early-stage trials with only upstream endpoints also play an important role and should be considered when evaluating the full spectrum of infertility trials. Finally, we only included published RCTs and hence, our results cannot be extrapolated to unpublished RCTs.

**WIDER IMPLICATIONS OF THE FINDINGS:**

The domination of RCTs on IVF calls for a reconsideration of other topics to be studied and a realignment of research priorities. The imbalanced geographic distribution of infertility trials raises questions about the generalizability of study results and equity in the distribution of healthcare resources. The prevalence of trials without registration or primary outcomes specified highlights the imperative to improve trial design and reporting quality. Encouragingly, the improving trial registrations suggest the enforcement of trial registrations from the journals is effective.

**STUDY FUNDING/COMPETING INTEREST(S):**

B.W.M. is supported by an NHMRC Investigator grant (GNT1176437). W.T.L. is supported by an NHMRC Investigator grant (GTN2016729). W.L.L. reports receiving a PhD scholarship from the China Scholarship Council. Q.F. reports receiving a PhD scholarship from Merck. B.W.M. reports receiving consultancy fees, travel support, and research funding from Merck; consultancy fees from Organon and Norgine; and stock ownership in ObsEva. T.D.H and S.L. are employees of Merck. W.T.L., W.L.L., and J.C. report no conflicts of interest.

**REGISTRATION NUMBER:**

PROSPERO CRD42024498624.

WHAT DOES THIS MEAN FOR PATIENTS? Randomized controlled trials (RCTs) are important for understanding how safe and effective the treatments are. Knowing about recent RCTs in infertility can help guide the design of future research. Trial registration (i.e. a public recording of the study’s design and goals) and a clear statement of the primary outcome (i.e. the main result being measured) are both vital for making research reliable. Here, we describe the characteristics, locations, and key methodological issues of infertility trials published in the last decade. We included RCTs in infertility that have reported clinical fertility outcomes, and we excluded secondary analyses or conference abstracts. Through manual and computer-aided data extraction, we found that over two-thirds of the 1425 infertility trials focused on *in vitro* fertilization, and nearly two-fifths of the trials used primary outcomes that were neither pregnancy nor live birth. The imbalanced distribution of the location of the trials was notable, with a small number of trials coming from countries with potentially large numbers of infertile couples. Half of the trials were not registered and a third did not state what the primary outcome was. However, trial registration and the reporting of primary outcomes have increased over time. Making sure trials are registered, clearly report their primary outcomes, and are conducted in a wider range of countries could improve the quality of infertility research and make it more useful for people around the world.

## Introduction

Infertility is defined as an unsuccessful pregnancy after 6–12 months of unprotected sex. Globally, it affects one in every six couples on average, while the prevalence can be as high as a quarter of the population, e.g. in the Western Pacific (World Health Organization, 2023). Infertility can be attributed to male or female factors, or both, and takes a profound psychological toll on both being affected by the condition. The inability to conceive naturally can evoke shame, guilt, frustration, and feelings of inadequacy, rendering individuals with infertility more vulnerable to suffering from discrimination and ostracism (Cui, 2010; Wang *et al.*, 2022).

Randomized controlled trials (RCTs) are a pivotal approach to address these challenges. They are the main source of evidence on the safety and effectiveness of interventions. For this to be possible, RCTs must pose questions that are pertinent to patients, be conducted with strong scientific rigour, and report findings in a transparent and accessible manner. Systematic reviews of a limited number of infertility trials revealed that under half of the trials had registration or specified primary outcomes at reporting (Farquhar and Marjoribanks, 2018; Rimmer *et al.*, 2022). Among those specified outcomes, only half used pregnancy or live birth as primary outcomes (Rimmer *et al.*, 2022). However, it remains unknown to what extent RCTs in infertility are conducted in such a manner, at a large scale. Understanding the strengths and limitations of RCTs from the recent past will help to pinpoint research gaps and prioritize the future research agenda. To obtain a holistic view of these issues in infertility, we herein provide an overview of RCTs in infertility published in the last decade for their characteristics, geographic distribution, and selected methodological issues.

## Methods

This is a systematic review of RCTs in infertility published from 1 January 2012 to 30 August 2023. A research protocol was designed and uploaded to OSF (https://osf.io/sgq6w) and the study was registered at PROSPERO (CRD42024498624) before the study was conducted.

### Identification of literature

We systematically searched Embase, Medline, and the Cochrane Central Register of Controlled Trials for RCTs in infertility published between 1 January 2012 and 30 August 2023 using a comprehensive search strategy (Supplementary Table S1). Eligible RCTs were those involving infertile women and reporting either biochemical pregnancy, clinical pregnancy, ongoing pregnancy, or live birth as outcomes. Trials which did not report these outcomes were not identified in the literature search. Full texts of included trials were reviewed to confirm that at least one of the four above-specified pregnancy outcomes was reported. We excluded conference abstracts, interim analyses, secondary analyses, or follow-up studies. We did not limit our search based on the language of the articles. Article screening was conducted by W.L.L. and J.C. independently.

To test whether the literature search and screening results were reliable and complete, we developed a validation set of infertility trials from Cochrane reviews. A detailed description of how the article set was constructed is provided in Supplementary File S1. In short, we started by searching for Cochrane systematic reviews focusing on female infertility published between 2012 and 2023. Among 339 Cochrane reviews identified, we manually selected 78 reviews for those that had reported pregnancy or live births. We then applied our eligibility criteria against the infertility trials cited by these reviews, which boiled down to 84 trials included in our validation set.

### Preparing data for extraction

We downloaded articles in portable document format (PDF) via Endnote, a reference managing software, using the ‘find full-text’ function (Clarivate, 2023). Articles not accessible through Endnote were manually obtained via university subscription or interlibrary loans.

Our data extraction process involved a semi-automated workflow combining text mining, the process of extracting data from unstructured natural language by software, and manual data extraction conducted by the first author. The full articles were subjected to text mining first, and the manual data extraction came into play when automated data extraction could not pin down a fixed language expression in which the data are embedded, e.g. sample size. This hybrid approach for data extraction has been used widely by other authors of systematic reviews (Gates *et al.*, 2021; Hill *et al.*, 2024). It serves as a sensitive, precise, and efficient alternative to data extraction solely by humans (Lajeunesse, 2016; Pham *et al.*, 2021).

To enable data extraction by computers, we parsed the articles in PDF format into the extensible markup language (XML), a readable and extractable format for computers. The parsing work was performed using Grobid, a machine-learning tool developed for parsing raw documents from PDF to structured XML files, which was specifically designed for academic articles written in English (Lopez, 2009, 2023). It has been used for over a decade in research with satisfying reliability (Miah *et al.*, 2022). The accuracy and precision consistently outperform other automated data extraction tools (Lipinski *et al.*, 2013; Meuschke *et al.*, 2023).

### Data extraction and validation

The table for recording extracted data was created before data extraction and piloted in 20 studies. It was then revised after discussions with all co-authors. For each type of data that was extracted with the aid of text-mining, we first constructed an algorithm designed to identify text patterns containing the desired data for extraction. We then manually extracted data from a dozen articles randomly selected to compare its results with the data extracted by the algorithm, after which the algorithm was adapted accordingly. This trial-and-error process was then repeated multiple times until the algorithm could accurately extract over 90% of the targeted data. For the articles where the algorithm identified the absence of the data (e.g. the blinding method was missing) or targeted data had no fixed pattern of expression (e.g. sample size or patient recruitment country), we complemented the text-mining approach by extracting data manually. For articles written in other languages, we first extracted available data from their English-language abstracts. If the data were not available in the abstract, we translated the full article into English using Google Translate (Pedrana *et al.*, 2019) and extracted data manually or asked for help from a native speaker. To confirm the reliability of the data extracted using this method, we randomly selected 50 articles and manually checked the accuracy of each data extraction.

### Downloading other metadata

To ascertain the retraction status of each article, we linked articles included in our database with Retraction Watch using the digital identifier of an object (DOI) (Retraction Watch, 2024). Using the same method, we downloaded other metadata including conflicts of interest and funding statements from Dimensions, a database of abstracts, citations, and research grants, chosen for its robust and comprehensive metadata (Hook *et al.*, 2018). The first author then manually ascertained whether the funding source was from the government or industry.

To understand how efficient the public research investment was across different countries, we used the quantity of publication of trials, regardless of the quality of trials, divided by gross domestic product (GDP) per country in 2023 as an indicator of research efficiency. The GDP data per country in 2023 were downloaded from the website of the United Nations (2022). Further, we used the number of trials per adjusted number of newborns (number of births divided by the total fertility rate of that country) as a proxy indicator to measure the adequacy of infertility trials conducted in each country. The number of newborns each year per country in 2023 was downloaded from Database Earth (Database Earth, 2023). The total fertility rate of each country was downloaded from the United Nations (2023).

### Statistical analysis

We used proportions and frequencies to describe the trend. All data extraction, statistical analyses, and graphs were performed using R software (R Core Team, 2013).

## Results

**Figure 1.**
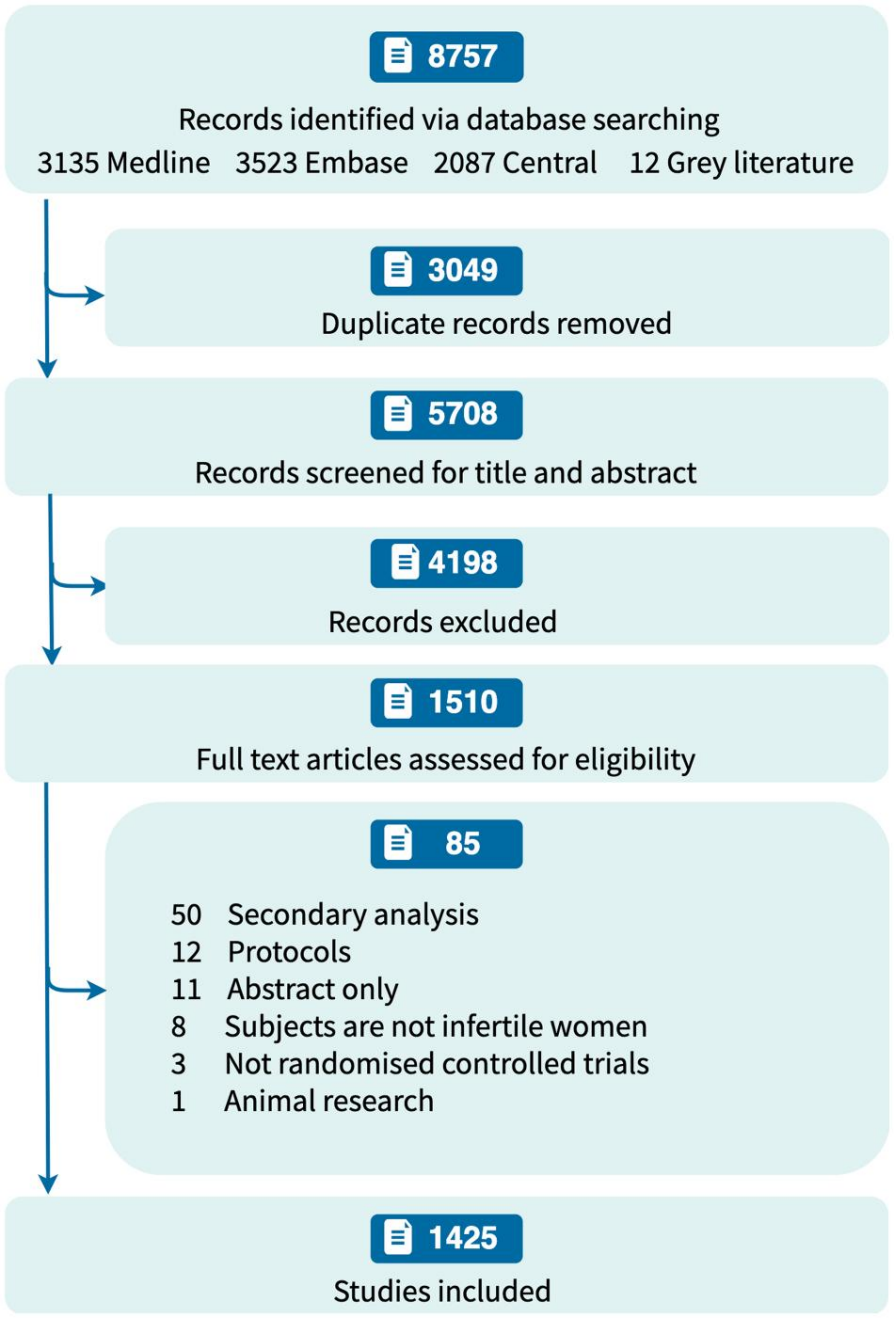
**Flowchart of the study**.

From the detected 8757 records, we identified 1510 RCTs by screening their titles and abstracts (Fig. 1). We further excluded 85 ineligible articles that were secondary analyses, conference abstracts, or non-RCTs after reading the full articles, resulting in 1425 RCTs included in our analysis.

### Manual checking of literature search and data extraction

We compared the identified 1425 RCTs against the validation set of infertility trials to assess the completeness of our literature search. Out of the 84 RCTs in the validation set, 77 (91.4%) were found in our 1425 RCTs. The reasons why our literature search missed the remaining seven RCTs are provided in Supplementary File S1. On a manual checking of 50 articles for the accuracy of extracting trial characteristics, the false negatives (e.g. the trial received funding, but our extraction indicated it did not) and false positives (e.g. a trial did not register, but our extraction indicated it did) were all at or below 8% for each type of data extracted. The detailed breakdown of manual checking of results by data type can be found in Supplementary Table S2.

### RCT characteristics

Of 1425 RCTs, the majority of the RCTs (93.3%, n = 1330) in our study were written in English (Table 1). The median sample size was 140 and it ranged from 18 to 2772, with 33.3% (n = 474) of the trials having fewer than 100 participants, while 4.3% (n = 62) of RCTs could not be ascertained for their sample size as the author only provided the number of oocytes or embryos randomized. Furthermore, parallel arm studies (99.3%), single-centre studies (82.4%), and studies with controls composed of routine care or no treatment (91.3%) were the most common study designs. Of all the trials, 46.8% (n = 668) did not provide information on whether and how the blinding was performed, while the most common type of blinding method was open-label studies which made up 19.8% of all trials.

**Table 1.** Baseline characteristics of infertility trials from 2012 to 2023.

Characteristics	Number of trials	Proportions
**Language**		
English	1330	93.3%
Chinese	67	4.7%
Other languages	28	2.0%
**Sample size**		
<100	474	33.3%
100–200	467	32.8%
200–500	294	20.6%
500–1000	85	6.0%
>1000	43	3.0%
Unclear	62	4.3%
**Study design**		
Parallel	1415	99.3%
Cross-over	8	0.6%
Factorial	2	0.1%
**Single or multicentre study**		
Single centre	1174	82.4%
Multiple centres	251	17.6%
**Blinding**		
Open label	282	19.8%
Single blind	239	16.8%
Double or triple blind	236	16.6%
Not described	668	46.8%
**Control group**		
Placebo	124	8.7%
Routine care or no intervention	1301	91.3%
**Funding**		
Declared not receiving any funding	1096	76.9%
Governmental	274	19.2%
Industry	55	3.9%
**Trial registration**		
Yes	744	52.20%
No	681	47.80%
**Intervention type**		
*In vitro* fertilization	992	69.6%
Ovulation induction	177	12.4%
Intrauterine insemination	151	10.6%
Surgery	69	4.8%
Lifestyle	16	1.1%
Tubal flushing	6	0.4%
Other interventions	14	1.1%
**Primary outcome number**		
Not specified the primary outcome	536	37.6%
1	629	44.1%
2	147	10.3%
3	58	4.1%
>3	55	3.9%
**Primary outcome category (among the trials with one primary outcome)**		
Hormone levels	34	5.4%
Oocyte or embryonic parameters	124	19.7%
Pregnancy	297	47.2%
Live birth	103	16.4%
Other outcomes	71	11.3%

Trials focusing on IVF made up 69.6% of all trials (n = 992). Ovulation induction and intrauterine insemination contributed to 12.4% (n = 177) and 10.6% (n = 151) of all trials, respectively, while the remaining 7.4% of trials included surgery, tubal flushing, lifestyle interventions, etc. Among trials reporting a primary outcome (n = 629), pregnancy of any stage as the primary outcome accounted for 47.2% (n = 297), while oocyte or embryonic parameters accounted for 19.7% (n = 124) and live birth accounted for 16.4% (n = 103) of the trials.

### Selected methodological issues

The majority of the trials declared that they did not receive funding from any source (76.9%), while the proportion of studies funded by government and industry was 19.2% and 3.9%, respectively. Among the top five countries producing the highest number of infertility trials, the proportions of funded trials were as follows: China (31.9%), Iran (27.1%), Egypt (4.3%), Turkey (0%), and India (8.5%). Sweden (72.7%) had the highest proportion of funded trials, followed by New Zealand (71.4%) and Denmark (66.7%) (Supplementary Fig. S1).

Unregistered trials made up 47.8% of all trials. Over time, the proportion of studies that were unregistered halved from 70.0% in 2012 to 34.6% in 2022. The proportion of trials adopting placebo and single-centre designs remained almost unchanged (Supplementary Fig. S2).

There were 536 trials (37.6%) that did not specify what the primary outcome was (Table 1). The proportion of trials specifying primary outcomes rose steadily from 49.5% in 2012 to 61.4% in 2022. A total of 260 (18.3%) of the trials had more than one primary outcome, which was often composed of a mix of various outcomes on effectiveness (e.g. number of oocytes retrieved, number of high-quality embryos, and clinical pregnancy rate, all as a primary outcome in a single RCT; data not shown).

### Geographic distribution of trials

**Figure 2.**
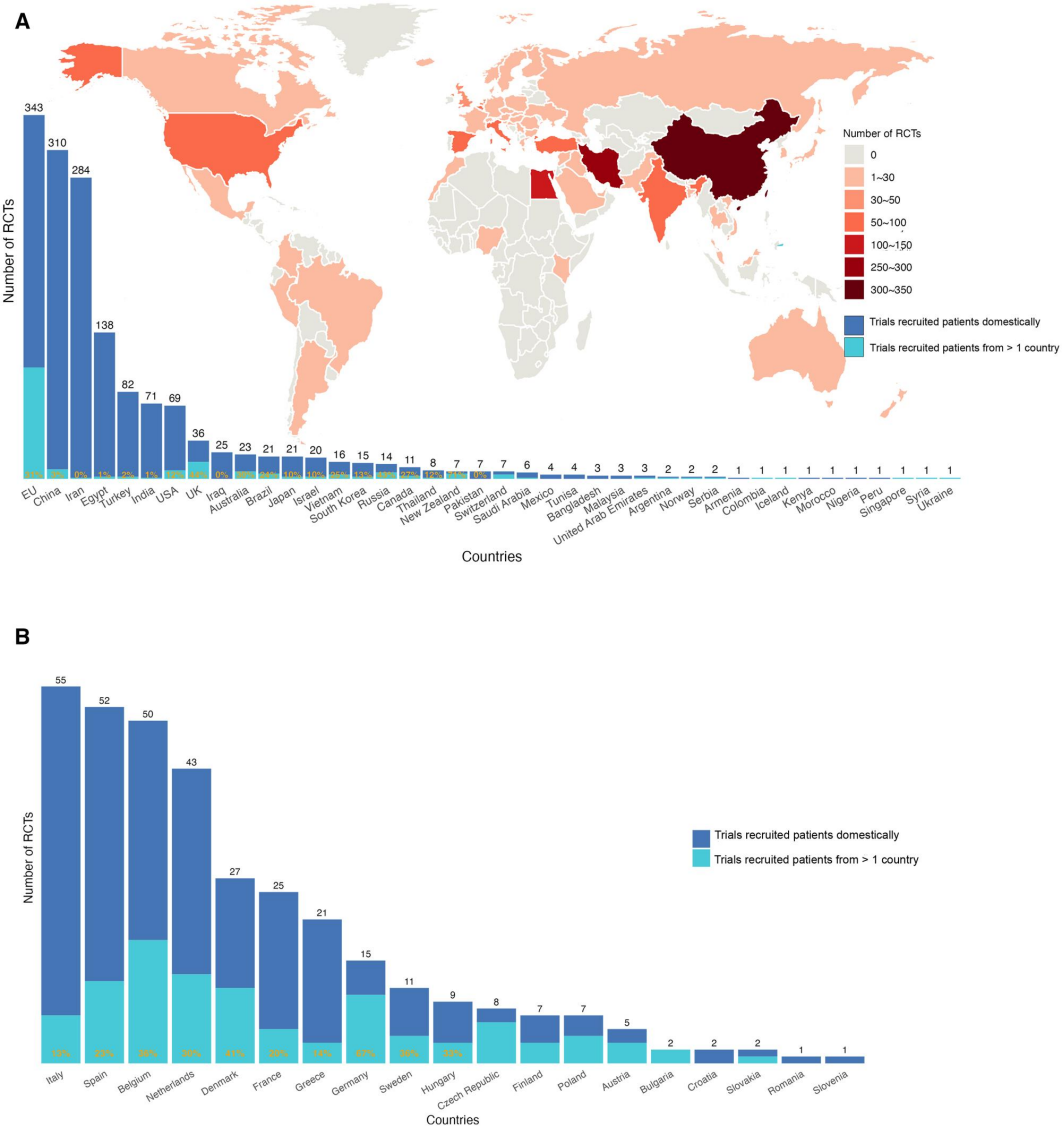
**The geographic distribution of trials in infertility published from 2012 to 2023.** (**A**) The overall trend in the number of trials, with European Union (EU) countries grouped collectively. (**B**) The trends within individual EU countries showing the contribution of each member state to the overall regional trend.

European Union (EU) countries were first grouped together, and then the trend was analysed across a breakdown of individual EU countries. Regarding the number of trials produced by each region (Fig. 2A), China was the top contributor (n = 310), followed by Iran (n = 284), Egypt (n = 138), Turkey (n = 82), India (n = 71), and the USA (n = 69). The number of trials published by the EU was 343, with Italy being the most prolific country within the EU (n = 55), closely followed by Spain (n = 52) and Belgium (n = 50) (Fig. 2B). Internationally collaborative trials, defined as trials recruited in different countries, were most common in the EU countries (31%), while this ratio was below 5% among the other top three most prolific countries (Supplementary Fig. S3).

**Figure 3.**
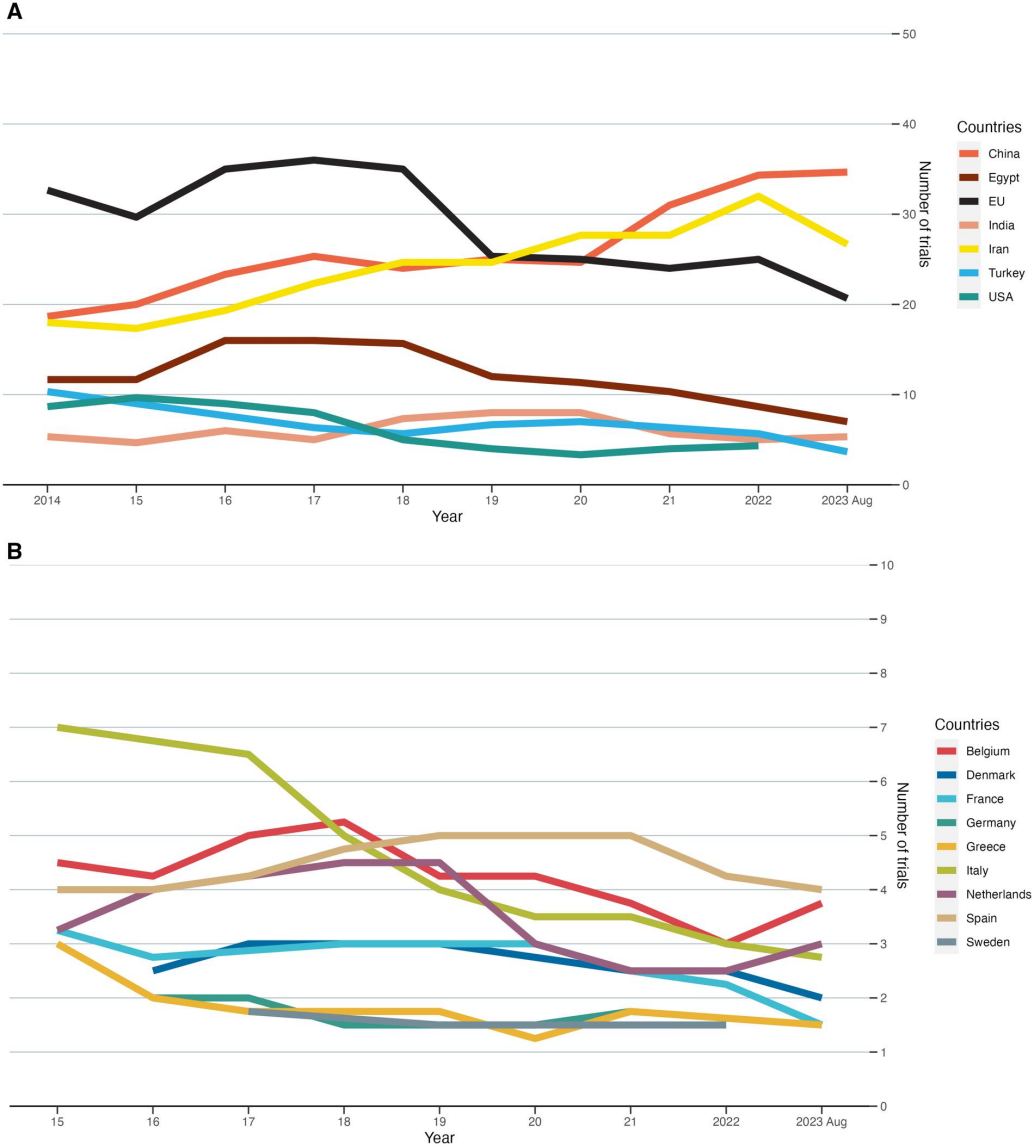
**The annual number of trials in infertility published by each country or region in the top seven most prolific countries (or regions).** (**A**) The number of trials in infertility by each country or region when European Union (EU) countries are grouped. (**B**) The number of trials in infertility by each country or region within EU countries. The lines represent the rolling average of three consecutive years.

Among the top seven most prolific regions, including the EU, China, Iran, Egypt, Turkey, India, and the USA, China and Iran saw the most rapid growth in the number of trials published each year, overtaking the EU as the top contributor in this field in 2018 (Fig. 3A). Egypt saw a mild decrease in the number of trials produced each year, while the number of trials published by the remaining regions remained stable over the years. Regarding EU countries, Italy held the top spot before 2017 but slumped since then, overtaken by Spain as the top contributor in 2022 (Fig. 3B).

Among 1425 RCTs, there were 16 retractions and four expressions of concern, of which 13 articles were from Egypt and three were from China, while the remaining four were from India, Iran, Italy, and Turkey. The list of retracted articles and their reasons for retraction are provided in Supplementary Table S3.

### Research output by GDP or newborns

**Figure 4.**
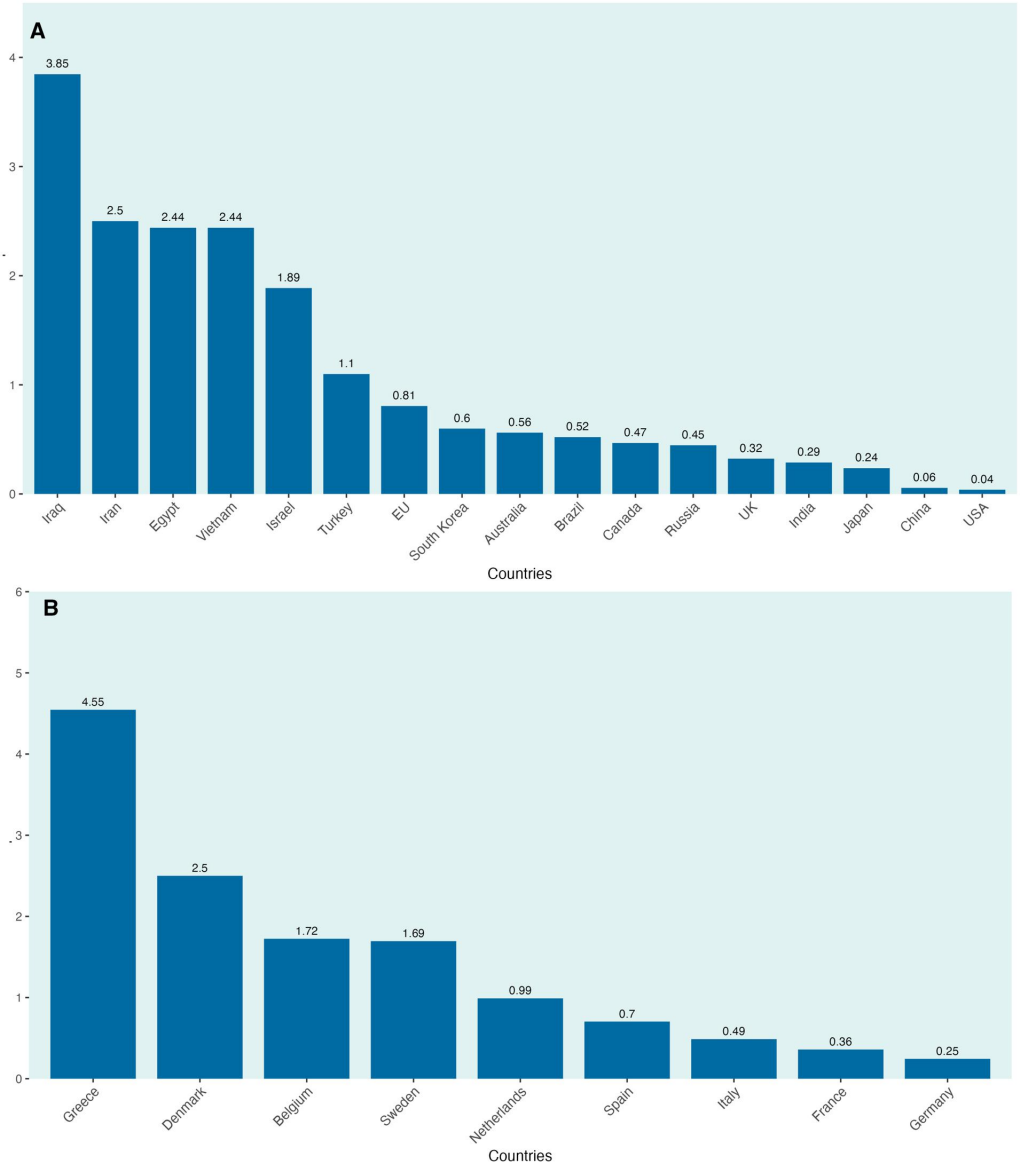
**The number of trials in infertility published per trillion gross domestic product (GDP) among the top 17 most prolific countries (or regions).** (**A**) The number of trials in infertility published per trillion GDP when European Union (EU) countries are grouped. (**B**) The number of trials in infertility published per trillion GDP within EU countries.

Regarding the number of publications in RCTs of infertility per trillion GDP, Greece ranked as the top among all regions at 4.6 (Fig. 4B); Iraq was the second, at 3.9 publications of RCTs per trillion GDP, followed by Iran and Denmark, both at 2.5 (Fig. 4A and B). While the USA and China had the highest GDP among all regions, both were at the bottom of this ranking, at 0.04 and 0.06 RCTs per trillion GDP, respectively.

**Figure 5.**
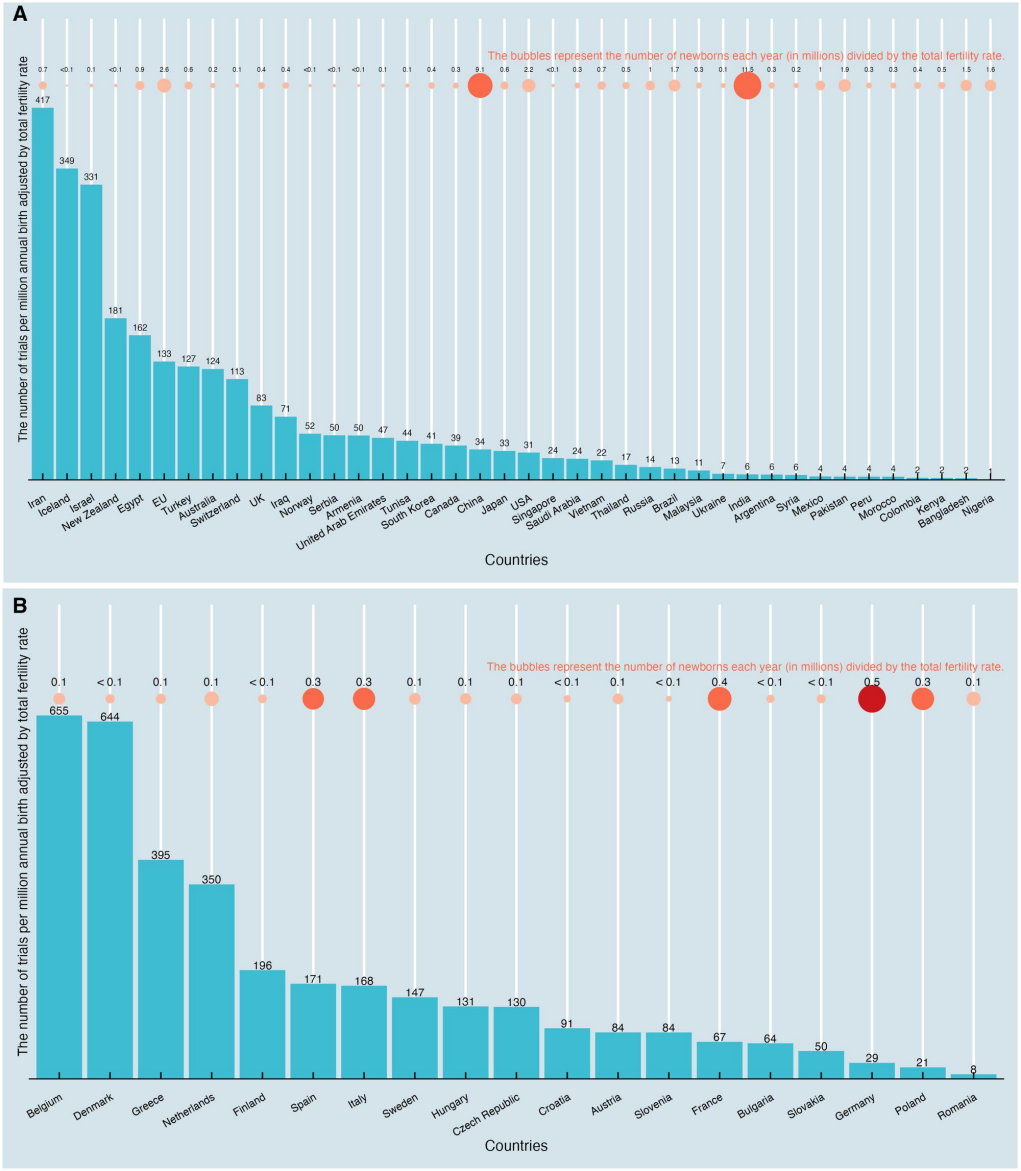
**The number of trials in infertility published per newborn each year in millions, adjusted by total fertility rate, in all countries (regions)**. (**A**) The number of trials in infertility published per newborn each year in millions when European Union (EU) countries are grouped. (**B**) The number of trials in infertility published per newborn each year in millions within EU countries.

Within EU countries, the number of publications per million newborns, adjusted by total fertility rate, was highest in Belgium (n = 655), with Denmark (n = 644) and Greece (n = 396) following (Fig. 5B). Outside the EU, Iran (n = 417) had the highest number of publications per adjusted annual newborns in millions (Fig. 5A). While India, Nigeria, and Pakistan had the highest fertility rates, their trials per adjusted million newborns were at the bottom of the ranking, with each having less than seven publications per adjusted million annual newborns.

## Discussion

In this overview of literature comprising 1425 infertility trials, we observed that nearly two-fifths of the trials used primary outcomes that were neither pregnancy nor live birth, while nearly 70% of all trials focused on IVF. Trials from China, Egypt, and Iran made up half of the global infertility trials, while trials from regions with the fastest-growing populations, such as India and Nigeria, were under-represented. In terms of methodological issues, half of the trials were not registered, and a third of the trials did not specify a primary outcome. Encouragingly, there has been a consistent rise in the trial registration rate and the number of trials specifying primary outcomes over time.

To our knowledge, this is the largest comprehensive overview of infertility trials published in the last decade. We provided a holistic overview of the current state and evolving trends of infertility trials globally, laying the groundwork for future-focused research questions on specific methodological issues, such as temporal changes in trial registration and the proportion of trials conducting interim analyses. Mapping such a landscape represents an important stepping stone towards addressing these questions and optimizing resources used for future research in infertility.

Prospective trial registration is a crucial measure for fostering research transparency and accountability. However, the registration rate, including prospective and retrospective trial registration, was not optimal in infertility trials. While we did not distinguish between prospective and retrospective registration in the trials, the low overall registration rate suggests that fewer than half were likely registered prospectively. This inadequate compliance with trial registration was also noted by others within and beyond the fertility field (Farquhar *et al.*, 2017a). The reasons for prospective registration deficiencies are multifactorial. They might be attributed to poor awareness among trialists, lack of organization and supervision of the trials, or inadequate enforcement by journals to which the manuscript is submitted. Lack of prospective registration warrants attention as unregistered trials have a higher risk of bias for random sequence generation, allocation concealment, and selective reporting, all of which undermine the reliability and validity of the trials’ results (Farquhar *et al.*, 2017b). Moreover, unregistered trials are less likely to be identified and included in secondary analyses, hence contributing to publication bias. Encouragingly, we saw the proportion of registered trials increased over time, thanks to the mandatory trial registrations required by the International Committee of Medical Journal Editors and enforced by journal editors (Zarin *et al.*, 2017). Other measures, such as the mandatory requirement for National Institutes of Health-funded trials to register and post-results at ClinicalTrials.gov, might also play a role in boosting prospective trial registration (Riley *et al.*, 2018). However, we cannot rule out that the rising trial registration was due to retrospective trial registrations, which offer no safeguard against selective reporting. To further improve prospective trial registrations, it is critical that there is concerted enforcement of prospective registration throughout the entire lifecycle of the trials made by national legislation, funding bodies, ethics committees, journal editors, and universities through self-regulation (Viergever and Li, 2015).

Our study showed an insufficient reporting of primary outcome, consistent with the observations in the fields of endometriosis and male infertility (Hirsch *et al.*, 2016; Rimmer *et al.*, 2022). Omissions of primary outcomes can hinder the interpretability, replicability, and synthesis of RCTs, which are crucial for informing clinical guidelines and decision-making. As demonstrated in our findings, there has been a mild but consistent increase in the proportion of trials specifying primary outcomes over time. This could be attributed to the Consolidated Standards of Reporting Trials (CONSORT) checklist, which requires authors to pre-specify the outcomes explored in the study and has been widely endorsed by journal editors. To improve the primary outcome reporting, a continued effort should be made to increase the endorsement and compliance of CONSORT.

With regard to the primary outcomes used, trials often set primary outcomes that are peripheral to pregnancy and live birth, including hormonal levels, and oocyte or embryonic parameters, in line with the findings by Rimmer *et al.*, 2022. The motives for choosing these intermediate outcomes are that they take a shorter time to observe and are easier to measure. Furthermore, selective reporting could be another cause, in which authors choose to report positive results over negative ones. In addition, using upstream endpoints other than live birth may be more suitable in certain scenarios. For example, when an intervention has a clear, proposed mechanism, testing that mechanism can be more efficient, cost-effective, and ethical before assessing its impact on live birth in large, expensive trials (Wilkinson and Stocking, 2021). While focusing on these upstream outcomes enables researchers to understand the underlying mechanisms behind infertility treatments, they might not always correlate directly with the ultimate goal of achieving a successful pregnancy or live birth. Core outcome sets in infertility have been developed and endorsed by the reproductive health community to encourage reporting of outcomes relevant to patients with infertility, even when these are not the primary outcome (Duffy *et al.*, 2020b, 2021). Future studies are needed to assess the barriers preventing the uptake of the core outcome set by trialists and researchers. Moreover, studies with multiple primary outcomes were also observed in our analysis. Having multiple primary outcomes can threaten a study’s validity if not handled properly, as it increases the risk of Type I errors and may lead to selective reporting. While we did not assess whether statistical methods were used to control Type I error inflation in these cases, it is unlikely that such adjustments were frequently applied.

We observed that infertility trials with sample sizes of fewer than 100 were common. Trials of smaller sample sizes have the advantages of shorter time to complete and lower cost to conduct, and sometimes they are the only option when the prevalence of the diseases is rare. While trials of smaller sample sizes can provide evidence conclusive enough for changing clinical practices, for most research questions on infertility, a large sample size is often needed when using pregnancy or live birth as a primary outcome, as a significant portion of infertility couples can conceive naturally in the absence of any treatment (Wang *et al.*, 2020). While we did not measure whether the sample size calculation was based on robust statistical reasoning, a review of sample sizes in infertility trials by Stocking *et al.* found that even the largest infertility trials lacked the power to detect plausible improvements in live birth rates (Stocking *et al.*, 2019). Compared to large trials, trials of smaller sizes are susceptible to publication bias, and carry a higher risk of having false-positive results and inflated treatment effects (Dechartres *et al.*, 2013). While the CONSORT and its extension have outlined specific items related to sample size calculations for the reporting of RCTs, future research is needed to assess to what extent trials have complied with the checklist and to identify the potential hurdles for compliance.

EU countries produced the highest number of trials, likely due to their high uptake of assisted reproductive technology (Hummel and Kettel, 1997; Berg Brigham *et al.*, 2013; Wyns *et al.*, 2021). This widespread acceptance of infertility treatments drives interest and demand for infertility research through clinical trials. Additionally, these countries benefit from well-established funding agencies and research infrastructure that actively support reproductive health studies. While the landscape of infertility trials should be applauded for its geographic diversity, we also observed a geographical disparity: a small number of infertility trials were conducted in countries with potentially a large number of infertile patients. For example, India represents almost 20% of the world’s population, yet only 5% of global infertility trials are conducted there (Worldometer, 2024b). Africa accounts for 18% of the global population, while the number of trials in Africa outside Egypt was little to none (Worldometer, 2024a). One rationale for promoting more clinical trials in these populous countries is that the disease characteristics and research agenda in these countries are different from others. For example, over three-quarters of infertility cases in sub-Saharan women are attributed to infection, while this rate in women worldwide is only 30% (Ombelet *et al*., 2008). On the other hand, while 70% of infertility trials are dedicated to IVF treatment, IVF in countries with a high total fertility rate is either woefully inadequate or prohibitively expensive (Chiware *et al.*, 2021). Nigeria has a fertility rate of 5.4, one of the fastest expanding populations in the world with a potentially large number of infertile couples, but the number of IVF clinics per million people was 0.46 (Ombelet and Onofre, 2019; TC Health 2024). This disparity underscores the need to align research resources with the pressing healthcare demands of rapidly growing populations (Duffy *et al.*, 2020a). In addition, there is a pressing need for more inclusive research efforts that address the specific needs of these under-represented regions, ensuring that the benefits of infertility research and treatments are globally accessible.

The number of trials per GDP can serve as a proxy for research prioritization, indicating which countries allocate more effort to infertility research irrespective of wealth. While high values can suggest that a country focuses more on infertility research relative to its economic capacity, this alone does not fully explain the patterns observed in our analysis. Greece, Iraq, Iran, and Egypt are among the top countries for infertility trials published per trillion GDP, yet they have unusually low proportions of funded trials. The reasons behind this paradox are unclear. There could be fewer organizations or governmental programs dedicated specifically to infertility research funding in these regions. There could also be a lower awareness of declaring funding resources in these countries.

Our study has limitations. The first is that while we optimized the algorithm for text-mining iteratively, there have been inaccuracies in data extraction. However, our manual checking revealed that the false-positive and false-negative rates were all at or below 8%. Using text mining for data extraction has been widely adopted by other authors of systematic reviews, particularly when the volume of studies makes manual extraction impractical (Thomas *et al.*, 2011; Pereira *et al.*, 2012; Walker *et al.*, 2022). Manually checking data extracted through text mining, as done in this study, is routinely performed to ensure the validity of the extracted data. Given the lack of a standardized framework for data extraction, a separate focused study could help further validate our approach. Secondly, while we extracted characteristics of the trials, some of the information was not granular enough to provide detailed insights into specific aspects of the research. For example, while we showed the proportion of trials that have used a placebo as a comparator, we did not ascertain whether the use of a placebo was plausible in the clinical context where the drug was under investigation. While trial registration status was ascertained, distinguishing between prospective and retrospective registrations was unfeasible due to the large volume of studies requiring manual extraction. Future research delving deeper into this question might provide more useful insights. Thirdly, we restricted our scope to trials reporting pregnancy outcomes, excluding those that reported only earlier endpoints. However, while trials reporting pregnancy outcomes are of primary interest to patients and carry significant clinical implications, early-stage trials also play an important role and should be considered when evaluating the full spectrum of infertility trials. Finally, the landscape is derived from trials that are published, suggesting the conclusions cannot be extrapolated to unpublished infertility trials.

## Conclusion

In a comprehensive overview of RCTs in infertility, we found a dominance of trials focusing on IVF, highlighting a need for reconsideration of other topics to be studied and a realignment of established research priorities. Trials from countries with potentially the largest number of infertile couples, such as India, were under-represented, raising a question about the equity of healthcare resources and generalizability of the results of trials. Inadequate trial registration rates and insufficient reporting of primary outcomes highlight the importance of improving trial design and reporting quality in this field. On a positive note, the consistent improvement in trial registrations suggests that the enforcement of trial registrations from the journals is effective and hence should be continued. These findings provide insights for the optimization of study design and prioritization of the research agenda.

## Supplementary Material

hoaf004_Supplementary_Data

## Data Availability

The data underlying this article will be shared on reasonable request to the corresponding author.
