# Supplementary material for: Trial characteristics, geographic distribution, and selected methodological issues of 1425 infertility trials published from 2012 to 2023: a systematic review
Source: Hum Reprod Open. 2025 Jan 24;2025(1):hoaf004. doi: 10.1093/hropen/hoaf004 (PMC11842059; doi:10.1093/hropen/hoaf004)

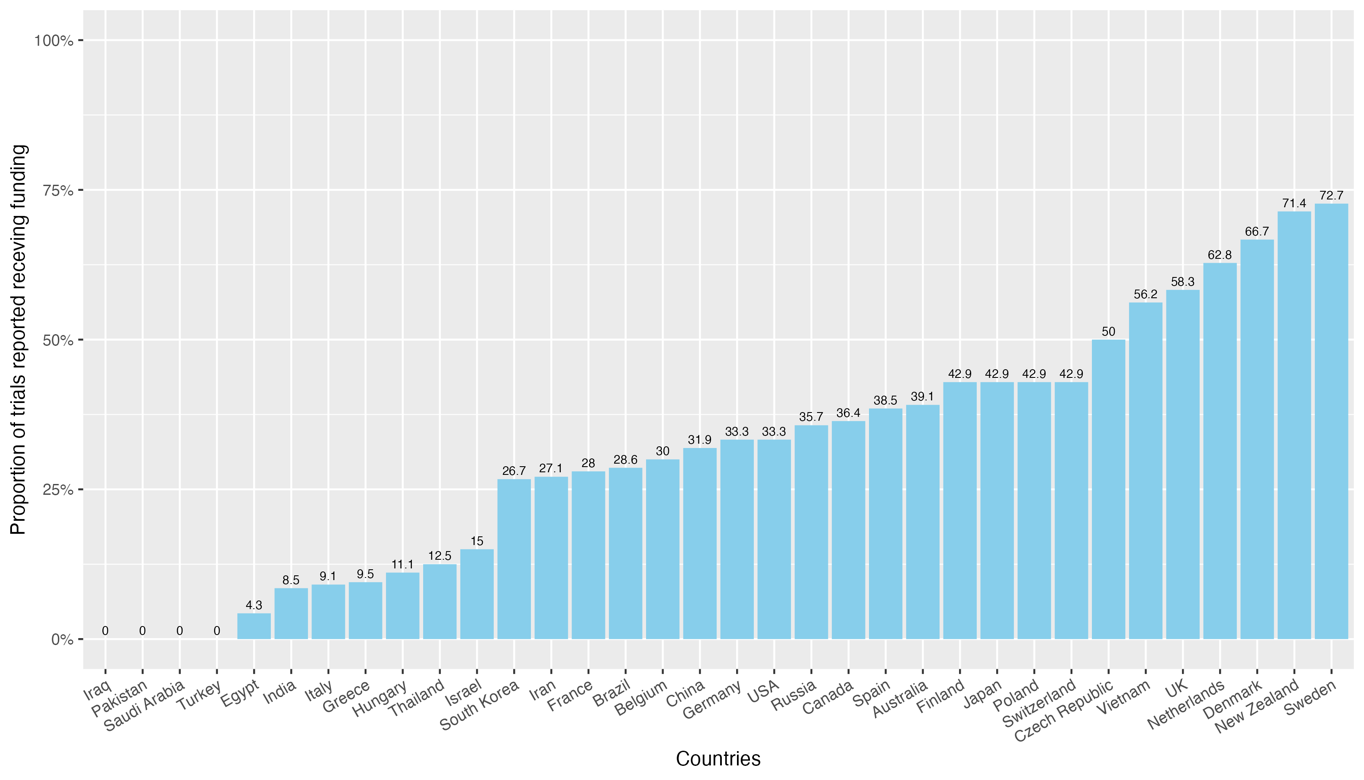
Supplementary Figure S1 The proportion of trials that declared receiving funding by countries


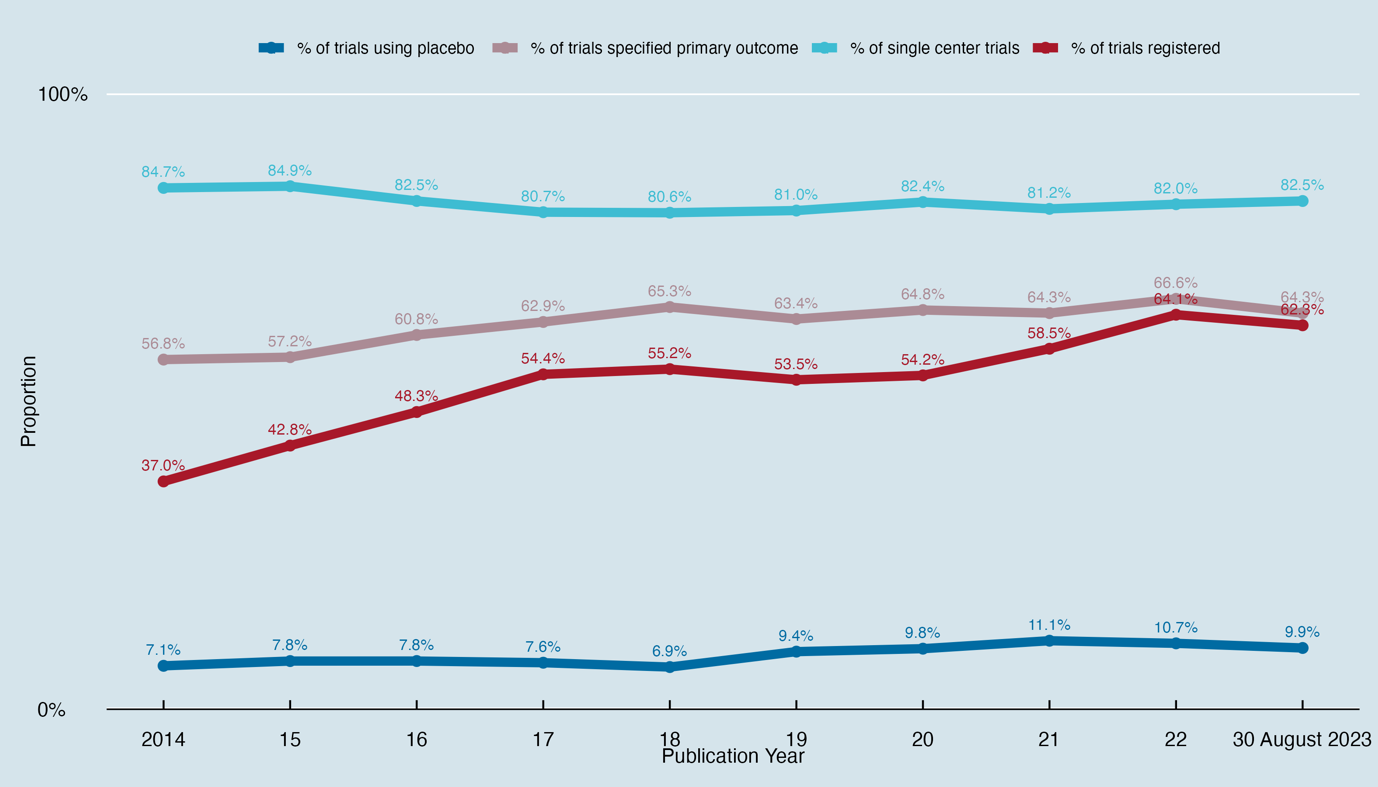
Supplementary Figure S2 The trend of selected trial characteristics over time

Supplementary Figure S3 The geographic distribution of trials in infertility published from 2012-2023, including the EU, a breakdown of EU countries and other countries. The lines represent the rolling average of three consecutive years.
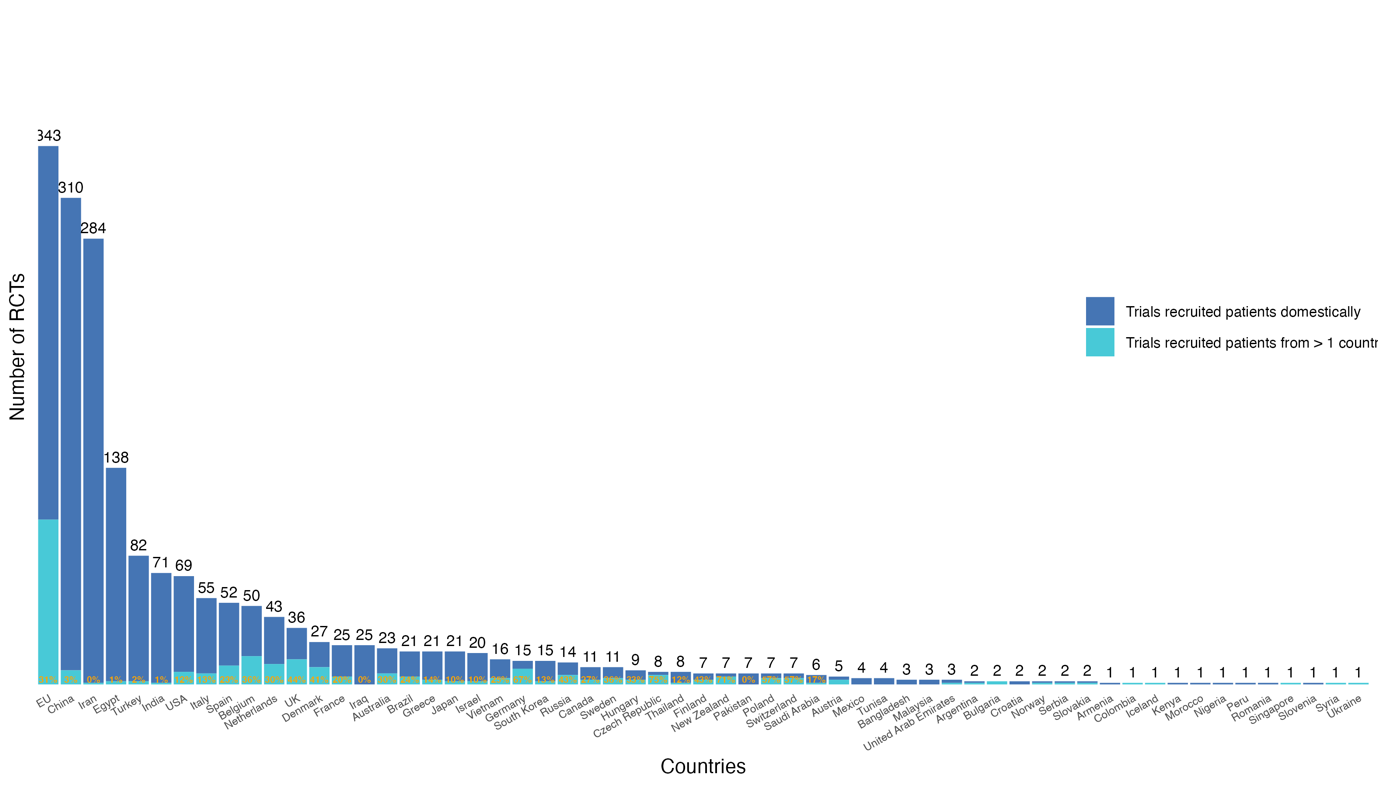

Supplement: hoaf004_Supplementary_Data [file hoaf004_supplementary_data.zip › Supplementary Figures combined.docx]
