# Supplementary material for: Trial characteristics, geographic distribution, and selected methodological issues of 1425 infertility trials published from 2012 to 2023: a systematic review
Source: Hum Reprod Open. 2025 Jan 24;2025(1):hoaf004. doi: 10.1093/hropen/hoaf004 (PMC11842059; doi:10.1093/hropen/hoaf004)
Supplement: hoaf004_Supplementary_Data [file hoaf004_supplementary_data.zip › HRO-24-0338-R2-SuppFileS1.docx]

# Supplementary File S1 The method for developing a validation set

Step 1. To develop the validation sets, we searched for the Cochrane reviews in infertility published from 1 January 2012 to 10 November 2023 using the following search strategy.We found 339 Cochrane reviews using the following search strategy

Inferti* or subfertil* or vitro fertili*ation or IVF or assisted reproduct* or intrauterine insemination or letrozole or clomiphene citrate or ovarian stimulation or ovulation induction or icsi or intracytoplas* sperm injection or superovulation or ovarian hyperstimulation or OHSS or luteal support or blastocyst or cleavage embryo or embryo transfer or oocyte pick* or oocyte retrieval or gonadotropin or embryo biopsy or frozen embryo transfer or FET or ovarian near/2 stimulation or implantation failure or embryo culture or embryo biops* in Cochrane CENTRAL webpage.

Step 2. Thenwe manually screened reviews that had a forest plot in reporting live birth, and 78 reviews were included. The DOI list of those 78 reviews are as follows.

10.1002/14651858.CD013063.pub2, 10.1002/14651858.CD013497.pub2, 10.1002/14651858.CD007876.pub2, 10.1002/14651858.CD011320.pub4, 10.1002/14651858.CD013233.pub2, 10.1002/14651858.CD007421.pub4, 10.1002/14651858.CD009517.pub4, 10.1002/14651858.CD003414.pub3, 10.1002/14651858.CD004378.pub3, 10.1002/14651858.CD004752.pub2, 10.1002/14651858.CD011537.pub3, 10.1002/14651858.CD005356.pub3, 10.1002/14651858.CD008528.pub3, 10.1002/14651858.CD005291.pub3, 10.1002/14651858.CD006359.pub3, 10.1002/14651858.CD006109.pub3, 10.1002/14651858.CD005070.pub3, 10.1002/14651858.CD004634.pub4, 10.1002/14651858.CD012692.pub2, 10.1002/14651858.CD001894.pub6, 10.1002/14651858.CD011184.pub3, 10.1002/14651858.CD009577.pub3, 10.1002/14651858.CD000317.pub4, 10.1002/14651858.CD008720.pub2, 10.1002/14651858.CD011809.pub2, 10.1002/14651858.CD011424.pub4, 10.1002/14651858.CD002808.pub3, 10.1002/14651858.CD006105.pub4, 10.1002/14651858.CD012396.pub2, 10.1002/14651858.CD003416.pub5, 10.1002/14651858.CD000099.pub4, 10.1002/14651858.CD003357.pub5, 10.1002/14651858.CD006919.pub4, 10.1002/14651858.CD012693.pub2, 10.1002/14651858.CD006942.pub3, 10.1002/14651858.CD003854.pub2, 10.1002/14651858.CD002811.pub4, 10.1002/14651858.CD003719.pub4, 10.1002/14651858.CD001750.pub4, 10.1002/14651858.CD008046.pub4, 10.1002/14651858.CD010290.pub3, 10.1002/14651858.CD006920.pub3, 10.1002/14651858.CD004829.pub4, 10.1002/14651858.CD009154.pub3, 10.1002/14651858.CD010042.pub2, 10.1002/14651858.CD006900.pub3, 10.1002/14651858.CD013240.pub2, 10.1002/14651858.CD010550.pub2, 10.1002/14651858.CD002249.pub5, 10.1002/14651858.CD009090.pub2, 10.1002/14651858.CD012375.pub2, 10.1002/14651858.CD007689.pub4, 10.1002/14651858.CD002125.pub4, 10.1002/14651858.CD006107.pub4, 10.1002/14651858.CD001122.pub5, 10.1002/14651858.CD001301.pub2, 10.1002/14651858.CD009452.pub2, 10.1002/14651858.CD004832.pub4, 10.1002/14651858.CD012856.pub2, 10.1002/14651858.CD010287.pub4, 10.1002/14651858.CD005996.pub4, 10.1002/14651858.CD001838.pub6, 10.1002/14651858.CD010461.pub3, 10.1002/14651858.CD012378.pub2, 10.1002/14651858.CD001502.pub4, 10.1002/14651858.CD011009.pub2, 10.1002/14651858.CD013505, 10.1002/14651858.CD003053.pub6, 10.1002/14651858.CD009749.pub2, 10.1002/14651858.CD011872.pub3, 10.1002/14651858.CD008189.pub3, 10.1002/14651858.CD010001.pub3, 10.1002/14651858.CD012650.pub2, 10.1002/14651858.CD007807.pub4, 10.1002/14651858.CD003718.pub5, 10.1002/14651858.CD003857.pub4, 10.1002/14651858.CD011345.pub3

Step 3.We manually identified the infertility trials included by the above reviews in Step 3 that had reported live births or ongoing pregnancies, which resulted in 84 infertility trials involving females and had reported live births or ongoing pregnancies. Here are the DOIs of the validation sets (n=84).

10.1007/s10815-014-0385-y, 10.1016/j.fertnstert.2012.12.043, 10.1093/humrep/dex231, 10.1007/s00404-014-3541-9, 10.1093/humrep/dew156, 10.1093/humrep/dey334, 10.1002/ijgo.12355, 10.1016/j.rbmo.2017.04.004, 10.1093/humrep/dev224, 10.1159/000363235, 10.1186/s12958-015-0069-1, 10.1093/humrep/dey268, 10.1056/NEJMoa1414827, 10.1016/j.ejogrb.2015.03.023, 10.1093/humrep/dev062, 10.1016/j.ejogrb.2013.03.002, 10.1093/humrep/dey262, 10.1007/s10815-016-0736-y, 10.1093/humrep/dew120, 10.1111/jog.13802, 10.1186/1477-7827-11-96, 10.1093/humrep/dev038, 10.1016/j.ejogrb.2020.03.003, 10.1111/1471-0528.14629, 10.1111/ajo.12168, 10.1016/S0140-6736(17)32406-6, 10.1016/j.fertnstert.2014.03.012, 10.1007/s00404-017-4604-5, 10.1016/j.repbio.2017.05.003, 10.1177/1933719116641764, 10.1056/NEJMoa1513873, 10.1016/j.fertnstert.2016.12.022, 10.1056/NEJMoa1705334, 10.1136/bmj.m2519, 10.1056/NEJMoa1703768, 10.1016/S0140-6736(18)32843-5, 10.1002/uog.14669, 10.1186/s43043-019-0001-2, 10.1007/s10815-017-0949-8, 10.1016/j.mefs.2018.05.002, 10.1177/1933719115602776, 10.1016/j.mefs.2016.06.006, 10.1111/cen.12294, 10.1093/humrep/dew268, 10.1016/j.fertnstert.2016.07.1096, 10.3109/09513590.2013.859242, 10.1016/j.rbmo.2015.04.013, 10.1071/RD13412, 10.1136/bmj.g7771, 10.1016/j.fertnstert.2013.09.035, 10.1016/j.ejogrb.2016.05.027, 10.1007/s40618-013-0021-1, 10.1016/j.ejogrb.2012.11.026, 10.1016/j.fertnstert.2013.09.010, 10.1016/j.fertnstert.2012.12.052, 10.3109/09513590.2013.813475, 10.1093/humrep/dew148, 10.1016/j.mefs.2013.05.012, 10.1111/j.1447-0756.2012.02072.x, 10.1093/humrep/deu263, 10.1001/jama.2017.7217, 10.1093/humrep/dew050, 10.1016/j.jmig.2018.10.013, 10.3892/etm.2015.2690, 10.1016/S0140-6736(21)00535-3, 10.1007/s00404-014-3397-z, 10.1016/S0140-6736(16)00231-2, 10.1093/humrep/dex227, 10.1056/NEJMoa1313517, 10.1080/09513590.2017.1332174, 10.1016/j.fertnstert.2016.05.022, 10.1016/S0140-6736(18)32989-1, 10.3109/09513590.2012.743020, 10.3109/09513590.2015.1101444, 10.1093/humrep/det108, 10.1186/s12958-015-0014-3, 10.4103/0974-1208.117174, 10.1056/NEJMoa1505297, 10.4137/CMRH.S14681, 10.3389/fendo.2018.00545, 10.1186/s12958-018-0343-0, 10.1056/NEJMoa1612337, 10.1142/S2661318219500063, 10.4103/jhrs.JHRS_134_16

Step 4. We compared the list of DOIs in the validation set with the RCTs included in my analysis. Among these 84 articles in the validation set, the following 7 were not identified in our database.

| DOI | Reasons for why it was incorrectly excluded from our study |
| --- | --- |
| 10.1016/j.mefs.2018.05. | The author stated the design was ‘randomized case control study’ in the abstract, whereas it should be randomized controlled study. |
| 10.3109/09513590.2012.743020 | The author stated the design was ‘randomized case control study’ in the abstract, whereas it should be randomized controlled study. |
| 10.1142/S2661318219500063 | The journal was not indexed in Pubmed or Embase, the two major databases that we searched |
| 10.4137/CMRH.S14681 | An honest error. It was accidently excluded by the reviewers at screening stage. |
| 10.4103/jhrs.JHRS_134_16 | No keywords such as biochemical/clinical/ongoing pregnancy were reported in the abstract. Therefore it was incorrectly filtered out at screening stage. |
| 10.14660/2385-0868-88 | The journal was not indexed in Pubmed or Embase |
| 10.1071/RD13412 | No key words such as IVF/infertile appear in the abstract. Hence it was accidently excluded. |
